# Redescription of *Hepatozoon felis* (Apicomplexa: Hepatozoidae) based on phylogenetic analysis, tissue and blood form morphology, and possible transplacental transmission

**DOI:** 10.1186/1756-3305-6-102

**Published:** 2013-04-15

**Authors:** Gad Baneth, Alina Sheiner, Osnat Eyal, Shelley Hahn, Jean-Pierre Beaufils, Yigal Anug, Dalit Talmi-Frank

**Affiliations:** 1 School of Veterinary Medicine, Hebrew University, P.O. Box 12, Rehovot, Israel; 2 Clinique Vétérinaire, 58, rue du Vigné bas, Calvisson, 30420, France; 3 Pathovet LTD, Yehosa Ben Hanania 81, Rehovot, 76391, Israel

**Keywords:** *Hepatozoon felis*, *Hepatozoon canis*, Domestic cat, Transplacental transmission

## Abstract

**Background:**

A *Hepatozoon* parasite was initially reported from a cat in India in 1908 and named *Leucocytozoon felis domestici*. Although domestic feline hepatozoonosis has since been recorded from Europe, Africa, Asia and America, its description, classification and pathogenesis have remained vague and the distinction between different species of *Hepatozoon* infecting domestic and wild carnivores has been unclear. The aim of this study was to carry out a survey on domestic feline hepatozoonosis and characterize it morphologically and genetically.

**Methods:**

*Hepatozoon* sp. DNA was amplified by PCR from the blood of 55 of 152 (36%) surveyed cats in Israel and from all blood samples of an additional 19 cats detected as parasitemic by microscopy during routine hematologic examinations. *Hepatozoon* sp. forms were also characterized from tissues of naturally infected cats.

**Results:**

DNA sequencing determined that all cats were infected with *Hepatozoon felis* except for two infected by *Hepatozoon canis*. A significant association (p = 0.00001) was found between outdoor access and *H. felis* infection. *H. felis* meronts containing merozoites were characterized morphologically from skeletal muscles, myocardium and lungs of *H. felis* PCR-positive cat tissues and development from early to mature meront was described. Distinctly-shaped gamonts were observed and measured from the blood of these *H. felis* infected cats. Two fetuses from *H. felis* PCR-positive queens were positive by PCR from fetal tissue including the lung and amniotic fluid, suggesting possible transplacental transmission. Genetic analysis indicated that *H. felis* DNA sequences from Israeli cats clustered together with the *H. felis* Spain 1 and Spain 2 sequences. These cat *H. felis* sequences clustered separately from the feline *H. canis* sequences, which grouped with Israeli and foreign dog *H. canis* sequences*. H. felis* clustered distinctly from *Hepatozoon* spp. of other mammals. Feline hepatozoonosis caused by *H. felis* is mostly sub-clinical as a high proportion of the population is infected with no apparent overt clinical manifestations.

**Conclusions:**

This study aimed to integrate new histopathologic, hematologic, clinical, epidemiological and genetic findings on feline hepatozoonosis and promote the understanding of this infection. The results indicate that feline infection is primarily caused by a morphologically and genetically distinct species, *H. felis*, which has predilection to infecting muscular tissues, and is highly prevalent in the cat population studied. The lack of previous comprehensively integrated data merits the redescription of this parasite elucidating its parasitological characteristics.

## Background

*Hepatozoon* species are apicomplexan parasites with a hematophagous arthropod final host and a vertebrate intermediate host. They are transmitted by ingestion of the final host containing mature oocysts by the intermediate host [1]. The gamont stage of the parasite is found in leukocytes or erythrocytes of the intermediate host and infects the final host during the blood meal. Additional transmission pathways have been described in some *Hepatozoon* spp. including intrauterine transmission and carnivorism of the intermediate host by an intermediate host of a different species [2-5]. More than 340 species of *Hepatozoon* have been described to date in amphibians, reptiles, birds, marsupials and mammals [1,6]. A *Hepatozoon* parasite was reported for the first time from the blood of a domestic cat in India by Patton in 1908 and named *Leucocytozoon felis domestici*[7]. The feline parasite was later transferred to the genus *Hepatozoon* and it was suggested that *Hepatozoon* parasites from the cat, jackal and hyena are indistinguishable from *Hepatozoon canis*, which infects dogs, due to the similarity in morphology of the gamont stage seen in the blood of these animals [8]. The classification of the *Hepatozoon* parasites found in domestic cats has thereafter been uncertain and most studies have carefully referred to *Heptozoon*-like parasites or *Hepatozoon* sp. without committing to a certain species [9-15]. With the advent of molecular techniques, PCR using genus-specific primers for *Hepatozoon* spp. was used to amplify *18S rRNA* gene DNA from the blood of a collection of wild and domestic animals including 2 cats from Spain. Although no parasites were morphologically described in the cat’s blood, the sequences from these cats were designated as *H. felis* and deposited in GenBank [14,16].

Domestic cat hepatozoonosis has been reported from several countries worldwide including: India, South Africa, Nigeria, the USA, Brazil, Israel, Spain and France [7,9,11,12,17-20]. Most studies have focused on reporting the detection of feline hepatozoonosis and almost no information has been published on its pathogenesis, transmission, life cycle and epidemiology. In that context, the aims of this study were to carry out a survey on domestic feline hepatozoonosis, characterize its causative agents genetically and morphologically in blood and tissues, and evaluate its possible transplacental transmission.

## Methods

### Collection of positive samples detected during routine laboratory evaluation

Anticoagulated blood in EDTA tubes from 19 domestic cats in which *Hepatozoon* sp. gamonts were detected by May Grunwald Giemsa-stained blood smear microscopy at the Hebrew University Veterinary Teaching Hospital (HUVTH) and at the private Pathovet Veterinary Pathology Laboratory in Israel during routine blood tests, were collected from 2002 to 2011 and stored at – 80 C.

### Cat survey

Blood samples were collected in EDTA tubes during 2010 and 2011 from multiple locations and sources in Israel. These included convenience sampling of cats whose *Hepatozoon* infection status was unknown from animal shelters in 5 cities in central Israel (Tel-Aviv; Jerusalem; Rehovot; Beit Dagan; Rishon Le-Zion), cats brought for routine spay to HUVTH, cats admitted to private veterinary clinics in 5 cities and villages in Israel (Haifa; Nahariya; Carmiel; Kfar Vradim; Yodfat) and to the to the HUVTH in central Israel whose samples were taken for routine diagnostic purposes. Serum samples were also collected from the same cats. Data collected on the cats included: sex, age, source of cats (e.g. shelter or private ownership), geographic location, indoors or outdoors access, and feline immunodeficiency (FIV) status as tested during this study.

### Cats tissues for evaluation of *Heptozoon* sp. infection

Formalin fixed paraffin-embedded tissues of 3 cats in which structures of *Hepatozoon* sp. meronts were detected by histopathology were included in the study. One cat was a patient at the HUVTH and detected antemortally as being parasitemic with *Hepatozoon* sp. It died with hepatitis and pancreatitis and was necropsied at the Kimron Veterinary Institute (KVI) pathology department. Fresh tissues from multiple organs were also collected from this cat at necropsy and stored at -80 C. A second cat was diagnosed with feline panleukopenia at the HUVTH and necropsied at the KVI following its death. Tissues including myocardial and skeletal muscle from a third cat diagnosed with *Hepatozoon* sp. infection in France [11] were submitted to the Hebrew University by its attending veterinarian.

Fetuses from shelter queens brought to the HUVTH for spaying and found to be pregnant during the neutering procedure were frozen at -80 C and if the queen blood was found to be positive for *Hepatozoon* sp. by PCR, fetal tissues were dissected using separate sterile scalpels for each tissue and submitted for *Hepatozoon* PCR in order to detect possible intrauterine transmission.

### DNA extraction

DNA from the blood of cats was extracted using the illustra blood genomicPrep Mini Spin Kit® (GE Healthcare, Buckinghamshire, UK) according to the manufacturer’s instructions. Samples from paraffin-embedded tissues were deparaffinized and DNA was extracted using the QIAamp DNA FFPE tissue kit® (QIAgen, Valencia, CA, USA) according to the manufacturer’s instructions. DNA from frozen tissues was extracted using the guanidine thiocyanate technique as previously described [21].

### PCR for the detection of *Hepatozoon* spp. and *Toxoplasma gondii*

A partial fragment of the *18S rRNA* gene of *Hepatozoon* spp. was amplified by PCR using primers Piroplasmid-F CCAGCAGCCGCGGTAATT and Piroplasmid-R CTTTCGCAGTAGTTYGTCTTTAACAAATCT [20]. The following conditions were used: 94°C 3 min, 35 cycles of [94°C 30 s, 64°C 45 s, 72°C 30 s] 72°C 7 min. PCR was performed using the Syntezza PCR-Ready High Specificity kit (Syntezza Bioscience, Israel). Positive *H. canis* control samples (5 μl DNA) from naturally infected dogs positive by blood smear and by PCR and sequencing of the PCR product, and negative controls were run with each PCR. This PCR protocol was used for all of the samples included in the study. Other sets of primers tried for amplification of a partial fragment of the *18S rRNA* gene such as the HEP-F and HEP-R primers [22] were less successful in amplifying samples from domestic cats with hepatozoonosis.

A second PCR assay was performed in some of the samples positive by the Piroplasmid PCR to amplify a larger segment (approximately 1400 bp) of the *18S rRNA* gene of *Hepatozoon* spp. for further phylogenetic analysis. This assay used primers HAM-1 F GCCAGTAGTCATATGCTTGTC and HPF-2R GACTTCTCCTTCGTCTAAG [16]. The amplification conditions for this reaction were: 95°C, 5 min; (34x [95°C 20 sec, 56°C, 30 sec, 72°, 90 sec]; 72°C, 5 min). Positive *H. canis* control samples (5 μl DNA) from naturally infected dogs positive by blood smear and by PCR and sequencing of the PCR product, and negative controls were run with each PCR reaction.

In order to rule out possible misdiagnosis of *Hepatozoon* tissue forms with *Toxoplasma gondii* cysts, a PCR for *T. gondii* was carried out on DNA extracted from tissues of the 3 cats in which *Hepatozoon* sp. meronts were detected by histopathology. PCR was performed to amplify a 529 bp fragment *T. gondii* repeat sequence using primers TOX4 CGCTGCAGGGAGGAAGACGAAAGTTG and TOX5 CGCTGCAGACACAGTGCATCTGGATT [23] as previously described [24].

### Sequencing and phylogenetic analysis

All PCR amplicons amplified from positive cats included in each part of the study were sequenced. The DNA products were sequenced using the BigDye Terminator v3.1 Cycle Sequencing Kit (PerkinElmer/Applied Biosystems) and an ABI PRISM 3100 Genetic Analyzer (Applied Biosystems). Sequences were evaluated with the ChromasPro software version 1.33 and compared to sequence data available from GenBank using the BLAST 2.2.9 program (http://www.ncbi.nlm.nih.gov/BLAST/). The *Hepatozoon* species identity found was determined according to the closest BLAST match with an identity of 97% -100% to an existing GenBank accession. Only samples that produced amplicons with a sequence compatible with a *Hepatozoon* sp. were considered positive for *Hepatozoon* and included accordingly in the study analysis.

A phylogenetic analysis, which included DNA sequences from the blood of 18 cats from the study, was carried out to compare these sequences to *Hepatozoon* spp. described in other animal hosts and in domestic cats and had previously been deposited in GenBank. Sequences were analyzed using the MEGA version 3.0 (http://www.megasoftware.net) and a phylogenetic tree was constructed by the Maximum-Likelihood, Minimum-Evolution and the Neighbor-Joining algorithms using the Kimura2-parameter model. Bootstrap replicates were performed to estimate the node reliability, and values were obtained from 1000 randomly selected samples of the aligned sequence data.

### Parasite morphology and sizes

The sizes of parasites found in blood smear and histologic specimens were measured by a manual micrometer and light microscope.

### FIV serology

Detection of antibodies for FIV in cat sera was performed using the Feline Immunodeficiency Virus Immuno Run Antibody Detection assay® (Biogal Galed Labs, Israel).

### Statistical analysis

Data was analyzed using the Chi-Square, Fisher’s Exact and the Mann-Whitney tests. A p value < 0.05 was considered statistically significant. The study was approved by the HUVTH research projects study evaluation committee.

## Results

### Evaluation of positive blood smear *Hepatozoon* spp. sample collection

All 19 cat samples identified by blood smear microscopy during 2002-2011 and collected at the HUVTH were positive by PCR for *Hepatozoon* sp. using the Piroplasmid primers followed by DNA sequencing.

### Cat survey

*Hepatozoon* sp. DNA was amplified from the blood of 55 out of the 152 (36.2%) surveyed cats whose *Hepatozoon* infection status was unknown using the Piroplsamid PCR protocol.

The sex of the cat was recorded for 146 of the 152 survey cats. Seventy-five were males (51.4%) of which 30 (40%) were PCR+ for *Hepatozoon* and 71 were female (48.6%) of which 24 (33.8%) were PCR+. Sex was not found to be significantly associated (p = 0.438) with positivity for *Hepatozoon* infection in the surveyed cats using the Chi square test. The age was known for 126 surveyed cats for which the mean age and standard deviation (SD) were 4.6 ± 4.2 years with a range of 4 months to 18 years. No significant association (p = 0.453) was found between age and *Hepatozoon* positivity using the Mann-Whitney test.

Information on access to the outdoors was available for 132 cats. Ninety-two (68.7%) of the cats were outdoors or spent a part of the day outdoors of which 45 (48.9%) were PCR+ for *Hepatozoon*, whereas 42 (31.3%) were strictly indoor cats of which only 4 (9.5%) were PCR+. A significant association (p = 0.00001) was found between outdoor access and *Hepatozoon* positivity using the Chi square test.

Of the 101 cats belonging to private owners, 39 (38.6%) were PCR+ for *Hepatozoon* and of the 51 non-privately owned cats, 16 (31.4%) were PCR+. No significant association (p = 0.475) was found between cat ownership status and *Hepatozoon* positivity using the Chi square test. Likewise, when dividing the cats to those who originated from northern Israel (from Haifa northward) to those who were from central Israel, 29 of 67 (43.3%) cats from northern Israel were PCR+ for *Hepatozoon* and 26 of 85 (30.6%) cats from central Israel were PCR+ with no significant association (p = 0.854) found between the cat geographic origin and *Hepatozoon* positivity using the Chi square test.

Sera was only available from 64 cats for FIV testing. Fifty-four of these cats (84.4%) were negative for FIV of which 22 (40.7%) were PCR+ for *Hepatozoon*, and 10 (15.6%) cats were positive for FIV of which 6 (60%) were *Hepatozoon* PCR+. No significant statistical association (p = 0.312) was found between FIV and *Hepatozoon* infections using the Fisher exact test.

### PCR on cat tissues

*Hepatozoon* sp. DNA was amplified from paraffin-embedded tissues of 3 cats using PCR with the Piroplsamid primers. The first cat was detected antemortally at the HUVTH as being parasitemic with *Hepatozoon* sp. and at necropsy followed by tissue histopathology, it was described to be cachectic with chronic hepatitis and chronic pancreatitis. *Hepatozoon* sp. meronts were detected by histopathology in the skeletal muscles (semi-membranosus muscle of the hind limb) and in the myocardium of this cat. PCR carried out on DNA of paraffin embedded tissues from both the semi-membranosus and myocardial muscles was positive and sequenced. In addition, *Hepatozoon* PCR of fresh tissues collected at necropsy was positive for the lingual muscle (tongue), diaphragm muscle, longissimus dorsi muscle, semi-membranosus muscle, myocardium, liver, pancreas, spleen, kidney, lung, mesenteric lymph node and bone marrow. *Hepatozoon* PCR was also positive from blood taken antemortally.

The second cat diagnosed with feline panleukopenia had severe necrotizing enteritis and pneumonia by histopathology. *Hepatozoon* sp. meronts were detected in its myocardium and lungs by histopathology and these two tissues were also positive for *Hepatozoon* by PCR and sequencing.

Tissues from the third cat were sent from France where it was reported to have feline leukemia virus (FeLV) infection, *Hepatozoon* sp. parasitemia, and forms compatible with *Hepatozoon* meronts in its muscular tissues by histopathology [11]. Both the myocardium and skeletal muscles were positive for *Hepatozoon* by PCR and sequencing in the current study.

Tissues from all the 3 cats positive for *Hepatozoon* by PCR were PCR-negative for *T. gondii*.

### Detection of *Hepatozoon* DNA in fetal samples

Fetuses from 3 *Hepatozoon* blood PCR + shelter queens brought to the HUVTH for spay, included in the survey and found pregnant during neutering were tested by PCR for *Hepatozoon* DNA using the Piroplasmid primers. Uteri from 2 cats contained 3 and 4 relatively developed fetuses, respectively, from which lungs, liver, spleen, skeletal muscle from a back limb, cardiac muscle, naval tissue and amniotic fluid were collected from each embryo. The third uterus contained 4 small less developed fetuses from which abdominal cavity organ material and amniotic fluid were sampled. None of the fetuses from the uterus with 3 large embryos were positive for *Hepatozoon* by PCR. However, one developed fetus from the uterus with 4 fetuses was positive in the lung and amniotic fluid, and one of 4 less developed fetuses from the third uterus was positive in the amniotic fluid. In total, tissues from 2 of 11 fetuses were positive for *Hepatozoon* sp. by PCR.

### Genetic identity and phylogenetic analysis GenBank accessions

A 358 bp fragment of the *Hepatozoon 18S rRNA* gene was amplified by PCR using the Piroplasmid primers from the blood and tissues of all cats considered infected in this study. DNA sequencing revealed that all positive cats, except for two, had a sequence whose closest match by BLAST, with an identity of 97%-100%, was an *H. felis* GenBank accession. Most sequences were 98%-100% identical to *H. felis* [GenBank:AY628681] (*H. felis* isolate Spain 2 [16]). The DNA sequences similar to *H. felis* included those obtained from the 55 positive survey cats, sequences from all cats from which paraffin embedded or fresh frozen tissues were tested, and sequences from 17 of the 19 cats included in the collection of samples identified by blood smear microscopy during 2002-2011. The *Hepatozoon* sequence obtained from the blood of the cat in which infection was detected antemortally and also in necropsy tissue was 99% identical to *H. felis* [GenBank:AY628681]. The sequence amplified from the paraffin-embedded myocardial tissue of the French cat with hepatozoonosis was also 99% identical to *H. felis* [GenBank:AY628681] as was the sequence amplified from the paraffin-embedded myocardial tissue of the local cat with panleukopenia, and a sequence amplified from the amniotic fluid of a fetus from a blood-positive queen. Only 2 cats had DNA sequences that matched *H. canis* and not *H. felis*. Samples 9617 and 9618 from the collection of positive blood smear spp. were 99% identical to *H. canis* [GenBank:EU289222] and other *H. canis* accessions. Although the piroplasmid primers can also amplify DNA from piroplasms such as *Babesia* spp., none of the amplified sequences were positive for *Babesia*, *Theileria* or *Cytauxzoon* spp.

Further investigation of the genetic identity of *Hepatozoon* parasites found in cats from this study was carried out using the HAM-1 F and HPF-2R primers which amplified a 1400 bp fragment of the *H. felis 18S rRNA* gene. Amplification of this large fragment of the *18S rRNA* gene was carried out for selected samples included in the phylogenetic analyses. In addition to the DNA sequences from cats, fragments of *H. canis* from local Israeli dogs were also amplified using the two sets of primers and sequenced in order to compare *H. felis* and *H. canis* from cats with a local *H. canis* from dogs. Details on *Hepatozoon* sequences from this study submitted to GenBank are included in Table 1.

**Table 1:** **Description of new *Hepatozoon *spp. sequences from the study deposited in GenBank**

**Source and sample number**	***Hepatozoon *species**	**Primers used for amplification of *18S rRNA *gene fragment**	**GenBank accession number**
Cat 9617	*H. canis*	HAM-1 F and HPF-2R*	KC138531
Cat 9618	*H. canis*	HAM-1 F and HPF-2R*	KC138532
Cat 8533	*H. felis*	HAM-1 F and HPF-2R*	KC138533
Cat 1	*H. felis*	HAM-1 F and HPF-2R*	KC138534
Dog 7243	*H. canis*	HAM-1 F and HPF-2R*	KC138535
Cat 9685	*H. felis*	Piroplasmid-F and Piroplasmid-R**	KC138536
Dog 6417	*H. canis*	Piroplasmid-F and Piroplasmid-R**	KC138537
Dog 8672	*H. canis*	Piroplasmid-F and Piroplasmid-R**	KC138538
Cat 9617	*H. canis*	Piroplasmid-F and Piroplasmid-R**	KC138539
Cat 9618	*H. canis*	Piroplasmid-F and Piroplasmid-R**	KC138540
Cat 1778	*H. felis*	Piroplasmid-F and Piroplasmid-R**	KC138541
Cat 8987	H. felis	Piroplasmid-F and Piroplasmid-R**	KC138542

* [16]; ** [20].

**Figure 1:**
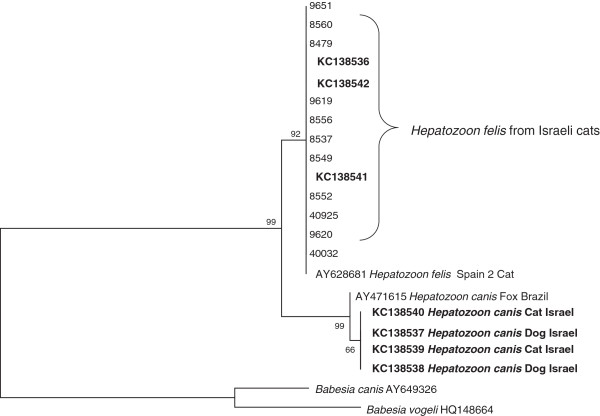
**Neighbor Joining *18S rRNA *Tree; A Neighbor Joining tree phylogram comparing 345 bp *18S rRNA *DNA *Hepatozoon *sequences from Israeli cats to other *Hepatozoon *GenBank deposited sequences with *Babesia vogeli *and *Babesia canis *as outgroups.** The GenBank accession numbers, species of infected animals and country of origin from which the sequences were derived are included for each sequence. New GenBank accessions derived from the present study are indicated in bold letters.

**Figure 2:**
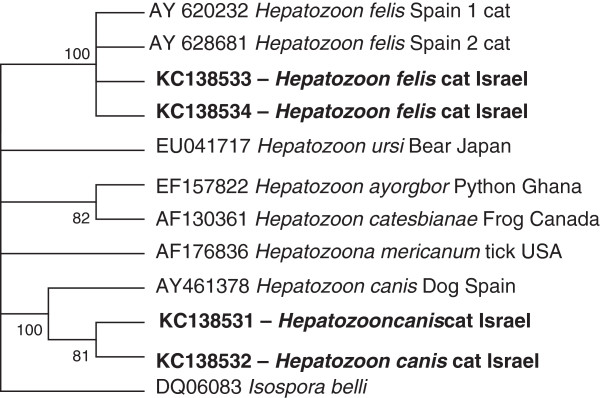
**Long *18S rRNA *segment Minimum Evolution Tree; A Minimum Evolution tree phylogram comparing 970 bp *18S rRNA *DNA *Hepatozoon *sequences from Israeli cats to other *Hepatozoon *GenBank deposited sequences with *Isospora belli *as outgroup.** The GenBank accession numbers, species of infected animals and country of origin from which the sequences were derived are included for each sequence. New GenBank accessions derived from the present study are indicated in bold letters.

A Neighbor Joining phylogenetic tree based on 345 bp from the shorter *18S rRNA* fragment amplified by the Piroplasmid primers (Figure 1) indicated that sequences from 14 Israeli cats detected as infected with *H. felis* clustered with *H. felis* [GenBank:AY628681] (*H. felis* isolate Spain 2), whereas the *H. canis* sequences found in 2 cats from the study clustered together with *H. canis* from dogs. Similar clustering was obtained also with the Maximum-Likelihood and the Minimum-Evolution algorithms. A Maximum Likelihood tree based on 970 bp from the longer *18S rRNA* fragment amplified by primers HAM-1 F and HPF-2R (Figure 2) also indicated that *H. felis* sequences from Israeli cats clustered together with the *H. felis* Spain1 and Spain 2 sequences. Likewise, *H. canis* from 2 Israeli cats clustered together with *H. canis* [GenBank:AY461378] from a dog in Spain. These were separated from *H. americanum* and *Hepatozoon* spp. reported from other animal hosts.

### Parasite morphology and sizes

**Figure 3:**
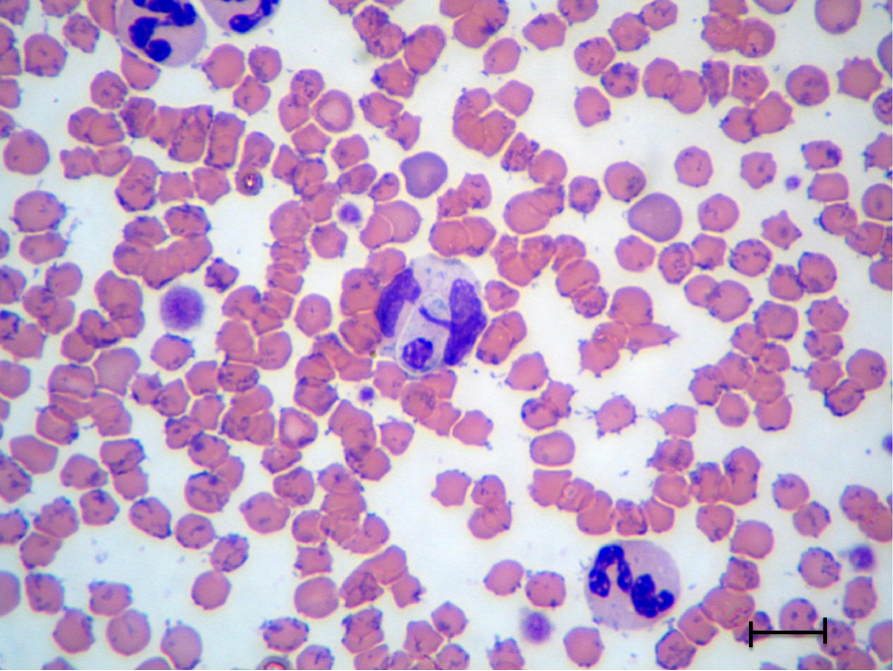
***Hepatozoon felis *gamont; A *H. felis *gamont in a monocyte from the blood smear of a cat co-infected with *Mycoplasma hemofelis*.** May Grunwald-Giemsa stain, Bar = 10 μm.

The life stages visible in the blood and tissues of cats positive for *H. felis* by PCR were visualized by microscopy and measured (Table 2). Gamonts observed in stained blood smears were located in the cytoplasm of neutrophils and monocytes sometimes compressing the lobulated host cell nucleus. Gamonts were elongated, enveloped by a visible membrane and possessed a round acentric nucleus (Figure 3). Some gamonts contained basophilic staining granules. In comparison to *H. canis* gamonts from dog blood, gamonts of *H. felis* were different due to their generally round nucleus, which is dissimilar to the more elongated horse-shoe shaped *H. canis* nucleus. They were also relatively shorter with a mean length of 10.5 μm compared with the *H. canis* gamont which is 11 μm long [25] and less conspicuous within the feline leukocyte than *H. canis* within its canine counterpart. The *H. felis* gamont was often hardly apparent within the host cell cytoplasm and almost concealed by its nucleus.

**Table 2:** **Sizes of *Hepatozoon felis *life stages**

**Stage**	**Source of sample**	**Mean size (μm) with SD**	**Shape index (length/width ratio)**	**Number measured**
Gamont	Cat blood	10.5 ± 0.6 x 4.7 ± 0.8	2.2	13
Gamont nucleus	Cat blood	4 ± 0.3 x 3.2 ± 0.5	1.2	12
Meront	Skeletal muscle; myocardium; lung	39 ± 5 x 34.5 ± 3.8	1.1	13
Meront capsule width	Skeletal muscle; myocardium; lung	1.4 ± 0.5	Not applicable	13
Merozoites	Skeletal muscle; myocardium; lung	7.5 ± 0.6 x 1.9 ± 0.3	3.9	14
Merozoite nucleus	Skeletal muscle; myocardium; lung	2.4 ± 0.5 x 1.6 ± 0.3	1.5	14

**Figure 4:**
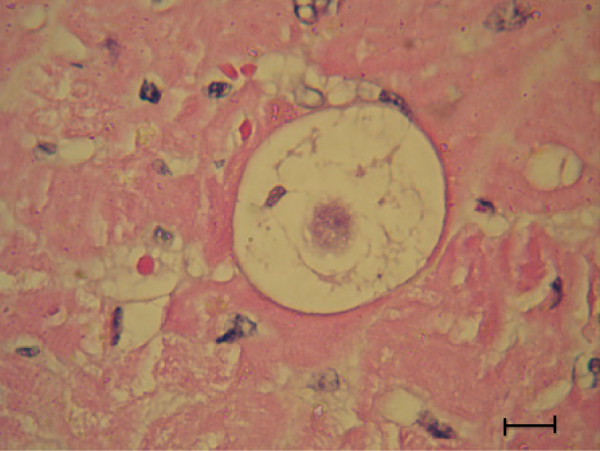
**Early *Hepatozoon felis *meront; An early *H. felis *meront in the myocardial muscle of a domestic cat.** Hematoxylin & Eosin stain, Bar = 10 μm.

**Figure 5:**
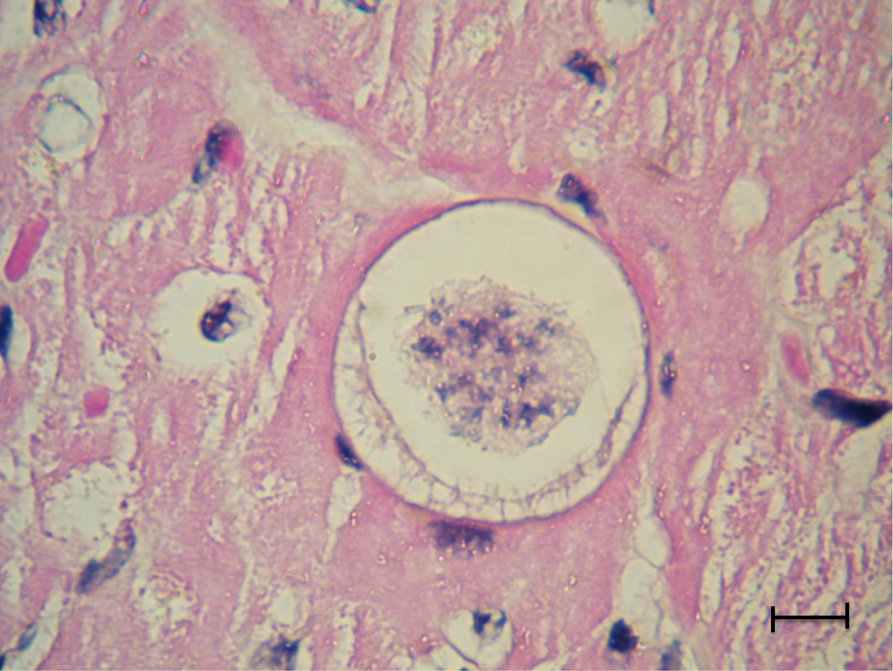
**Maturing *Hepatoon felis *meront; A maturing *H. felis *meront in the myocardial muscle of a domestic cat.** Note that merozoite nuclei are visible but distinct merozoites are not formed yet at this stage. Hematoxylin & Eosin stain, Bar = 10 μm.

**Figure 6:**
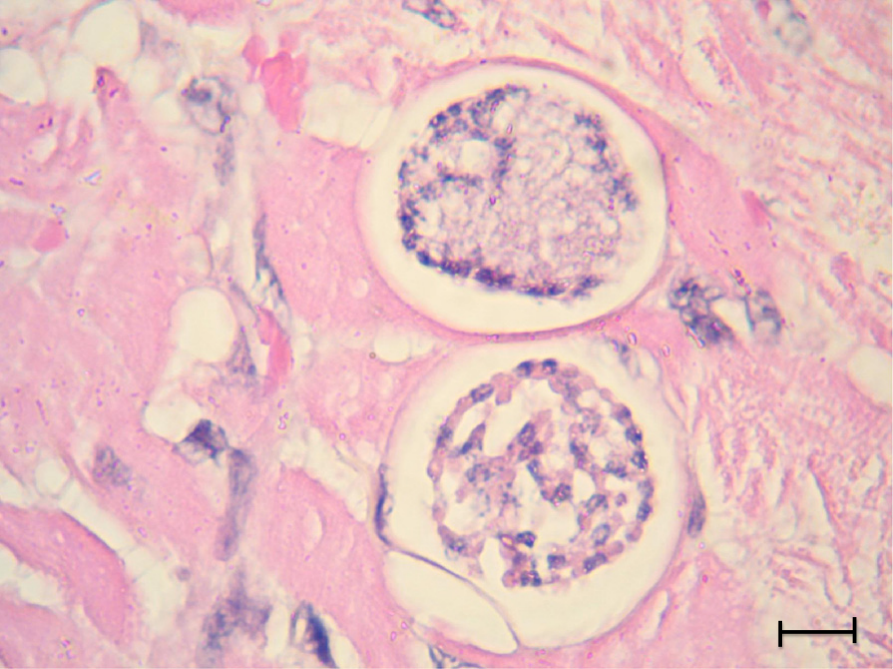
***Hepatozoon felis *meronts; Two maturing *H. felis *meronts in the myocardial muscle of a domestic cat.** Individual merozoites with separate nuclei are visible in the lower meront. Hematoxylin & Eosin stain, Bar = 10 μm.

**Figure 7:**
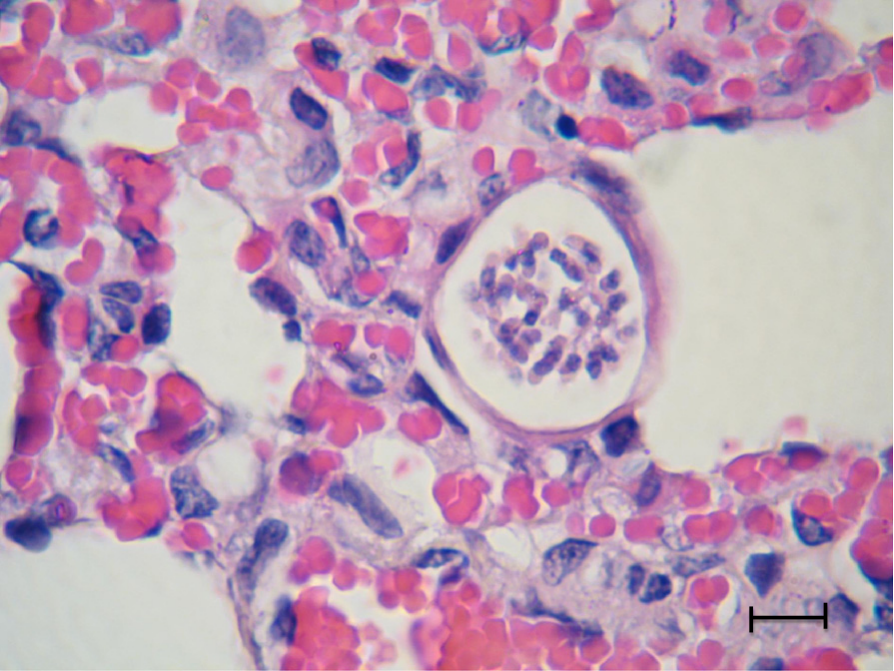
**Mature *Hepatozoon felis *meront in lung.** A mature *H. felis* meront in the lung of a cat with pneumonia and panleukopenia. Note the individual nucleated separated merzoites and thick external capsule. Hematoxylin & Eosin stain, Bar = 10 μm.

**Figure 8:**
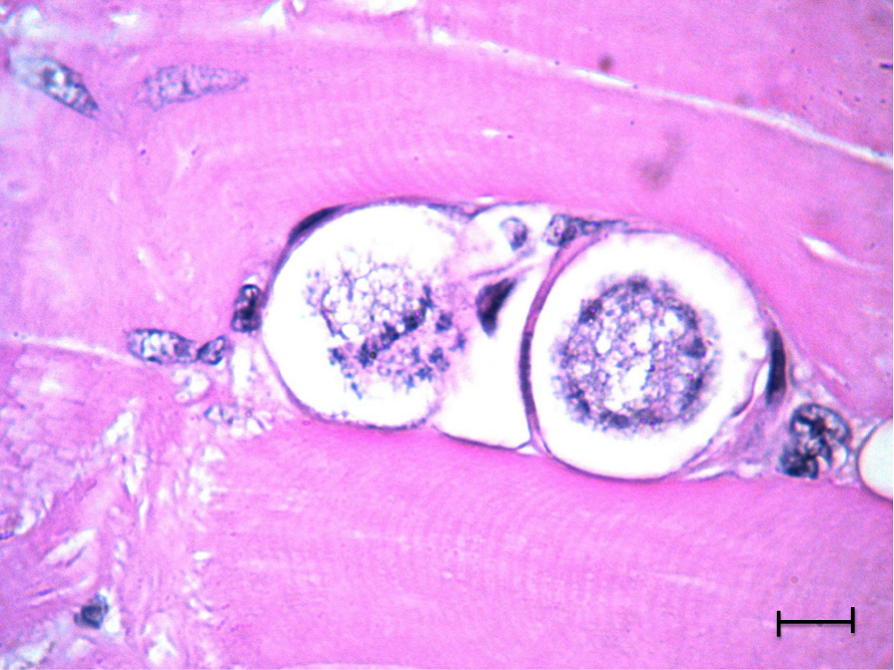
***Hepatozoon felis *meronts in striated muscle.** Two maturing *H. felis* meronts in the semi-membranosus skeletal muscle. No host inflammatory cells are visible in the tissue adjacent to the parasites. Hematoxylin & Eosin stain, Bar = 10 μm.

The *H. felis* meront is round to oval with a mean length of 39 μm by 34.5 μm and surrounded by a thick membrane separating it from the surrounding tissue (Table 2). The early *H. felis* meronts contain amorphous material without obvious zoites (Figure 4), and as they mature they form nuclei (Figure 5), which develop further into distinct intact long merozoites with a rectangular nucleus that assumes the whole width of the merozoite (Figures 6 and 7). The *H. felis* merozoites are dispersed within the meront without an obvious pattern of arrangement. Early *H. felis* meronts appear to have thinner capsules that widen and become thicker as the meront matures. No apparent inflammatory response was found associated with the presence of intact meronts in muscular tissues (Figure 8).

The *H. felis* meront is larger in size than the *H. canis* meront which is 30.6x 28.9 μm [26], found in muscular tissues such as the myocardium and skeletal muscles, unlike *H. canis* which infects hemolymphoid and parenchymal tissues, but not muscles. Furthermore, the *H. felis* meront does not form the typical wheel spoke shape of the *H. canis* meront with merozoites arranged in a circle along the meront circumference around a central core [26].

## Discussion

Although hepatozoonosis of domestic cats was initially reported in 1908, the same year when the type species *Hepatozoon muris* was described from a laboratory rat and its life cycle described [27], it has almost been overlooked since and little has been published on its pathogenesis. This study aimed to integrate new histopathologic, hematologic, clinical, epidemiological and genetic findings on feline hepatozoonosis and promote the understanding of this infection. The results clearly indicate that feline infection is primarily caused by a morphologically and genetically distinct species, *H. felis*, which has predilection to infecting muscular tissues, and is highly prevalent in the cat population studied. The lack of previous comprehensively integrated data merits the redescription of this parasite elucidating its parasitological characteristics.

Surveys of *Hepatozoon* sp. infection in domestic cats describe variable rates of infection in different areas. A study of myocardium specimens from 100 cats brought to necropsy in Israel found that 36% of cats harbored cardiac *Hepatozoon*-like meronts [9]. Interestingly, this was the same rate of infection found in Israel almost 40 years later in the current study, indicating that feline hepatozoonosis is not a new emerging infection in this country. Studies using PCR detection from Spain have shown diverse prevalence rates with 0.6% in one study [16], 16% in a cat colony from Barcelona [14], and 4% in cats from the Barcelona area [20]. A comparative study carried out in several districts of Bangkok, Thailand, where both canine and feline hepatozoonosis are prevalent, has reported a high infection rate of 32% in 300 cats by PCR. A positive association was found between the rates of infected dogs and cats in the same districts and *18S rDNA* sequences from cats and dogs were closest to *H. canis*[28]. These findings encouraged the authors to hypothesize that *H. canis* was the cause of both canine and feline infections. However, the genetic analyses in this study were probably made before the sequences designated as *H. felis* were deposited in GenBank [16]. Therefore, the identity of some feline sequences may have been misinterpreted. A study from Brazil evaluated 200 blood samples from Sao Luis in Brazil and found only one cat infected with a *Hepatozoon* sp. which clustered with *H. felis* on a phylogenetic analysis [15].

The lack of association between age and infection found in the study supports the possibility of transplacental transmission and kittens being born already infected. It may also suggest intensive exposure at a young age. The highly significant association found between infection and access to outdoors suggests the possibility of transmission by arthropod vectors, such as fleas, ticks or mites, which are common ectoparasites of cats globally, or by carnivorism as described for a number of *Hepatozoon* spp. [2-5]. So far, no arthropod vector has been described for *H. felis*. Other *Hepatozoon* spp. have been demonstrated to be transmitted by fleas, ticks, mites, lice, mosquitoes and sandflies [1] and it is therefore expected that *H. felis* would also be transmitted by an hematophagous arthropod. The existence of more than one route of transmission for *H. felis* is also optional as other *Hepatozoon* spp. have been shown to be transmitted both by arthropod vectors and by additional routes, for instance *H. canis* is transmitted by the tick *Rhipicephalus sanguineus* as well as transplacentally [3,29], and *H. americanum* is transmitted by the tick *Amblyomma maculatum* and by carnivorism [5,30].

The level of parasitaemia is usually low in feline hepatozoonosis with less than 1% of the neutrophils and monocytes containing gamonts [10]. A survey from Thailand found that parasitemia was detected by light microscopy of blood smears in only 0.7% of 300 cats, while 32% were positive by sensitive PCR [28]. Furthermore, none of the blood smears from 100 Israeli cats of which 36% were positive for cardiac *Hepatozoon* sp. meronts, had evident gamonts [9]. This study also found a higher infection rate in the myocardium of apparently healthy cats compared to sick cats [9]. Feline hepatozoonosis seems to be mostly sub-clinical as a high proportion of the population is infected with no apparent overt clinical manifestations. The seven cats described in a case series of feline hepatozoonosis suffered from various other infections including FIV, FeLV and hemotropic mycoplasmosis [10]. Although no significant association was found in the present study between FIV infection and *Hepatozoon* infection, this could be due to the small number of samples included, as proportionally, more FIV+ cats were infected with *Hepatozoon* than FIV negative cats. FIV or FeLV infections have also been described in conjunction with feline hepatozoonosis in other studies [11,14]. It is therefore probable that *Hepatozoon* infection may escape control by the immune-system in immune-suppressed cats, allowing the intensification of parasitemia and increasing the likelihood of detection by blood smear microscopy [10,11]. The reason for not testing the cats in the study for FeLV infection is the relatively low prevalence of this infection in local Israeli cats [31].

Feline hepatozoonosis is associated mostly with infection of muscle tissues. *Hepatozoon* sp. meronts have been reported in the muscles of domestic cats with hepatozoonosis [9,11], and elevated activities of the muscle enzyme creatine kinase were found in the majority of cats with hepatozoonosis in a retrospective study of this infection [10]. The genetic and morphologic findings of this study clearly showed that it is *H. felis* which infects myocardial and skeletal cat muscles, and not another *Hepatozoon* sp. However, other tissues such as the lungs were also infected with meronts, and PCR detected the presence of the parasite’s DNA in hemolymphoid organs such as the spleen, bone marrow and lymph nodes, and in the liver, pancreas, and kidney, as well as in fetal lungs and amniotic fluid. No substantial inflammatory response surrounding meronts was seen in the muscles examined in this study, as well in the other reports [9,11]. This is in agreement with the generally sub-clinical nature of this infection. Infection of muscle tissues by *Hepatozoon* sp. has also been reported in wildlife felids and carnivores where *H. felis* or closely related species are responsible for myositis and myocarditis [32-35]. *Hepatozoon americanum* infection of dogs and wildlife in the USA also has a predilection to muscle tissue, like *H. felis*, differing substantially from *H. canis* in dogs, which is found mostly in the hemolymphoid tissues and does not directly infect muscle. However, *H. americanum* induces the formation of muscle cysts which are much larger than *H. felis* meronts, are composed of concentric layers of muco-polysaccharide material surrounding a core zoite in a formation described as “onion skin” cysts, and elicit severe and painful pyogranulomatous myositis following merogony [24,36,37].

The phylogenetic placement of *18S rRNA Hepatozoon* sequences amplified from domestic cats in this study revealed that both *H. felis* and *H. canis* infect cats in Israel, although *H. felis* is by far more common. Israeli *H. felis* sequences are indistinguishable from those reported from Spain and Brazil, and closely related to those reported in wildlife felids and carnivores from India, Korea, Japan, Tanzania, Brazil and Argentina [15,16,33-35,38-40]. It is obvious that *H. felis* clusters away from *H. canis* and other *Hepatozoon* species, and is responsible for infection of domestic cats and likely also of other carnivores.

It is most likely that *H. felis* is the predominant species of *Hepatozoon* that infects domestic cats and wild felids globally. Its wide geographic distribution could be due to transmission by some ubiquitous vector such as a common flea, mite or tick species, or to highly successful alternative routes of transmission such as transplacental transmission or carnivorism of a yet unknown wildlife intermediate host. *Hepatozoon canis* has been shown to spread rapidly in a young dog shelter population, and it is possible that *H. felis* may also spread rapidly under similar conditions [41].

## Conclusions

Merging of morphologic and genetic findings on *H. felis* from multiple tissues and blood of the same cats, in conjunction with a broad-based epidemiological study, facilitated detailed characterization and redescription of this species in its intermediate host, the domestic cat. Further studies are needed to elucidate its definitive host, likely an arthropod vector, and other transmission pathways with transplacental transmission as a probable option.

## Competing interests

The authors declare that they have no competing interests.

## Authors’ contributions

GB designed the study, analyzed data wrote the manuscript; AS collected samples, performed PCR and analyzed data; SH performed and analyzed the histopathology; JPB contributed samples and assisted in writing the manuscript; YA analyzed collected samples and blood smears; DTE performed PCR on histopathological specimens, performed genetic analyzes and assisted in writing the manuscript. All authors read and approved the final version of the manuscript.
